# A Simple and Effective Way to Study Executive Functions by Using 360° Videos

**DOI:** 10.3389/fnins.2021.622095

**Published:** 2021-04-12

**Authors:** Francesca Borgnis, Francesca Baglio, Elisa Pedroli, Federica Rossetto, Giuseppe Riva, Pietro Cipresso

**Affiliations:** ^1^IRCCS Fondazione Don Carlo Gnocchi Onlus, Milan, Italy; ^2^Department of Psychology, Catholic University of the Sacred Heart, Milan, Italy; ^3^Applied Technology for Neuro-Psychology Lab, Istituto Auxologico Italiano, Istituto di Ricovero e Cura a Carattere Scientifico, Milan, Italy; ^4^Faculty of Psychology, eCampus University, Novedrate, Italy

**Keywords:** executive functions, assessment, eye tracker, EEG, machine learning, 360° environments, neurological disease

## Abstract

Executive dysfunctions constitute a significant public health problem due to their high impact on everyday life and personal independence. Therefore, the identification of early strategies to assess and rehabilitate these impairments appears to be a priority. The ecological limitations of traditional neuropsychological tests and the numerous difficulties in administering tests in real-life scenarios have led to the increasing use of virtual reality (VR) and 360° environment-based tools for assessing executive functions (EFs) in real life. This perspective aims at proposing the development and implementation of Executive-functions Innovative Tool 360° (EXIT 360°), an innovative, enjoyable, and ecologically valid tool for a multidimensional and multicomponent evaluation of executive dysfunctions. EXIT 360° allows a complete and integrated executive functioning assessment through an original task for EFs delivered *via* a mobile-powered VR headset combined with eye tracker (ET) and electroencephalograms (EEG). Our tool is born as a 360°-based instrument, easily accessible and clinically usable, that will radically transform clinicians’ and patient’s assessment experience. In EXIT 360°, patients are engaged in a “game for health,” where they must perform everyday subtasks in 360° daily life environments. In this way, the clinicians can obtain quickly more ecologically valid information about several aspects of EFs (e.g., planning, problem-solving). Moreover, the multimodal approach allows completing the assessment of EFs by integrating verbal responses, reaction times, and physiological data (eye movements and brain activation). Overall, EXIT 360° will allow obtaining simultaneously and in real time more information about executive dysfunction and its impact in real life, allowing clinicians to tailor the rehabilitation to the subject’s needs.

## Introduction

The executive dysfunctions in psychiatric and neurological pathologies constitute significant public health problems due to their high impact on personal independence, ability to work, educational success, social relationships, and cognitive and psychological development (Goel et al., 1997; Green et al., 2000; Diamond, 2013). Consequently, the assessment and the rehabilitation of these deficits must be early and adequate (Van der Linden et al., 2000). However, the evaluation of executive functions (EFs) represents a challenge due not only to the complexity of the construct (Stuss and Alexander, 2000) but also to the methodological difficulties (Chaytor and Schmitter-Edgecombe, 2003; Barker et al., 2004).

In the following paragraphs, we will briefly analyze the complex construct of EFs and the main tools for their evaluation.

### Executive Functions: A Complex Construct

Chan and colleagues described EFs as “an umbrella term comprising a wide range of cognitive processes and behavioral competencies which include verbal reasoning, problem-solving, planning, sequencing, the ability to sustain attention, resistance to interference, utilization of feedback, multitasking, cognitive flexibility, and the ability to deal with the novelty” (Chan et al., 2008). Impairments in EFs may have adverse effects in performing instrumental activities of daily living (IADL) including preparing meals, managing money, shopping for groceries or personal items, doing housework, and using a telephone (Chevignard et al., 2000; Fortin et al., 2003; Diamond, 2013). Interestingly, the ability to perform IADL appeared to relate to a person’s quality of life and feelings of personal well-being (Gitlin et al., 2001).

### Standard and Ecological Neuropsychological Tests

Standard paper-and-pencil neuropsychological tests have some difficulties in predicting what occurs in patients’ everyday life (Shallice and Burgess, 1991; Klinger et al., 2004; Słyk et al., 2019) and seem inadequate in terms of sensitivity or specificity. An ecological assessment appeared crucial to understand how executive deficits affect daily functioning (Burgess et al., 2006). For this reason, clinicians and researchers paid attention to develop tests capable of evaluating the different executive functioning components in real-life scenarios, such as the Multiple Errands Test (Shallice and Burgess, 1991; Alderman et al., 2003). The evaluation in real-life settings provided a more accurate estimate of the patient’s deficits than within laboratory conditions (Rand et al., 2009). Still, it showed further limitations, such as long times, high economic costs, difficulty of organization, and poor controllability of experimental condition or applicability in patients with motor deficits (Bailey et al., 2010).

All these problems have led to the increasing use of technological tools and virtual reality (VR) for an ecologically valid evaluation of EFs in real life.

### Virtual Reality

Virtual reality enables designing and developing realistic spatial and temporal scenarios, situations, or objects that, reproducing daily life conditions, allow an ecologically valid evaluation of EFs (Campbell et al., 2009; Bohil et al., 2011; Parsons, 2015).

VR-based tools allowed clinicians to observe their patients while performing everyday executive tasks in ecologically controlled environments (e.g., supermarkets, kitchens) (Climent et al., 2010; Maggio et al., 2018, 2019a,b).

Several research showed that assessment in these real-life scenarios provides a more accurate estimate of the patient’s impairment, showing difficulties invisible to traditional measurements (Rand et al., 2009; Armstrong et al., 2013; Cipresso et al., 2013a; Parsons, 2015). Finally, several studies showed the feasibility of VR-based tool as assessment for EFs in neurological [e.g., traumatic brain injury, Parkinson’s disease (PD), and multiple sclerosis (MS)] and psychiatric (i.e., schizophrenia and obsessive-compulsive disorder) populations (Josman et al., 2009; Raspelli et al., 2011; Aubin et al., 2018; Liu et al., 2019; Słyk et al., 2019).

In recent years, some authors used 360° environments (immersive photographs or videos) delivered *via* smartphones for presenting neuropsychological stimuli (Serino et al., 2017). The 360° technology belongs to the “virtuality continuum” of Milgram (Milgram and Kishino, 1994) in which stimuli are presented in a space between real and virtual, “mixed reality,” where the extremes may co-exist producing new experiences. This technology allows participants to be immersed in everyday scenarios that they experience from a first-person perspective. Moreover, it enables for sequential focusing upon several elements and portions of the environment at different times. Two studies conducted, respectively, with PD and MS showed that the 360° technology might play a role in ecological EFs’ assessment (Serino et al., 2017; Realdon et al., 2019). For example, patients with PD took longer to provide a correct interpretation of the scene proposed, gave significantly more detailed descriptions of the scene, and appeared more prone to distractor interference. These findings are in line with Luria’s view (Luria et al., 1966), suggesting that this test can capture deficits in active visual perception. Investigating patients’ eye movements during the interpretation of the proposed scene could be particularly interesting since deficits in active visual perception could reflect a disorganized scanning gaze’s activity (Luria et al., 1966). Thus, it would be interesting to integrate this technology with other portable devices, such as eye tracker (ET).

Several studies have tried to integrate the assessment of EFs by identifying new indices and non-verbal response using ET and electroencephalograms (EEG).

### Eye Trackers

Eye tracker allows for tracking and recording online the eye movements such as fixations or saccadic and antisaccadic movements. The combination of VR advantages (e.g., natural movements, controlled environment) and ET allows answering many research questions in a radically innovative way. ET allows constantly measuring the eyes’ displacements within the monitor screen’s spatial working area (Cipresso et al., 2013b). In particular, it will enable calculating the subject gaze’s direction in the 3D environment, defining regions of interest in 3D space and fixation time of each region (Clay et al., 2019). Over the years, several studies showed that antisaccade (AS) eye movements obtained with an AS task (AST) can be considered a good indicator of executive dysfunction (e.g., inhibition control) in patients with neurological disease and in elderly subjects (Mirsky et al., 2013). Firstly, Mirsky and colleagues showed that AST is a sensitive marker of executive dysfunction and frontal lobe structure in cognitively and functionally healthy elders (Mirsky et al., 2013). Results showed a correlation between the percentage of correct AS responses and neuropsychological measures of executive functioning (e.g., set-shifting, inhibition, and fluency task). Moreover, a significant relationship appeared between the severity of AS impairment and the degree of structural alterations in frontal inhibitory control network in healthy elders. Thus, AST is sensitive to subtle frontal lobe dysfunction that may indicate an increased risk of future cognitive decline.

Other studies showed the same good relationship between the AS responses and neuropsychological measures of EFs in clinical populations (Hutton, 2008; Heuer et al., 2013) that typically showed executive impairments, such as dementia (Boxer et al., 2006) and schizophrenia (Hutton et al., 2004; Levy et al., 2004). Recently, Ferreira and colleagues showed that patients with MS have higher error rates and prolonged latency than controls in the AS parameters (Ferreira et al., 2018). Thus, AST may be a selective and independent measure to investigate inhibitory control in MS. In the same year, Ouerfelli-Ethier and coworkers demonstrated AST’s suitability as a cognitive marker of EFs in aging and PD populations (Ouerfelli-Ethier et al., 2018). AS performance was a good predictor of decision-making and visual memory abilities in all samples and of visual search performance in clinical ones.

### Electroencephalographic Biosignals

The crucial role of EEG in the study of neural functionality is well known (Abiri et al., 2019). For example, EEG allows analyzing, monitoring, and recording electrical activity and any functional anomalies affecting the prefrontal cortex and related associated cortico-subcortical circuits (e.g., amygdala), responsible for EFs (Raz, 2000; Burke and Barnes, 2006). Several studies showed that slower oscillations (between 4 and 8 Hz) within the theta frequency band over the frontal cortex are associated with the engagement of neural resources involved in executive function processes. Moreover, a greater synchronization (power) within the theta frequency during a working memory task is associated with better performance (Onton et al., 2005). Other research showed a correlation between frontal brain activation and other EFs such as arithmetic strategy use (De Smedt et al., 2009) and complex non-verbal problem-solving (Doppelmayr et al., 2005). Over the years, EEG has been used for estimating the complexity of executive dysfunction in clinical populations (Yu et al., 2016). For example, Teramoto and colleagues showed that the decrease in resting-state functional connectivity between the frontal and parietal cortex, especially in the left side, is related to executive dysfunction in PD (Teramoto et al., 2016). Moreover, EEG is used to evaluate the neurophysiological effects of EFs’ training, such as increased task-related synchronization of the theta frequency band over frontal electrode sites (Best et al., 2019).

The traditional multi-electrode EEG systems have long been used only in hospitals and laboratories (Maskeliunas et al., 2016). Recent technological advances allowed developing portable, wearable, and wireless EEG headsets (Malechka et al., 2015; Suresh et al., 2015; Abiri et al., 2019). These inexpensive EEG devices with few channels and/or dry electrode made feasible to take this interesting technology outside of the laboratory into real-world environments. In other words, these affordable and easy-to-use devices, such as Emotiv EPOC and Neurosky, allow monitoring individuals’ EEG signals in their natural environment (Poltavski, 2015; Maskeliunas et al., 2016; Sawangjai et al., 2019). Interestingly, this innovative mobile EEG could be interesting for the evaluation of EFs in everyday settings and situations.

Given the promising results in using EEG and ET to assess executive functioning, their integration could provide several advantages. Firstly, clinicians could obtain simultaneously and in real time more non-verbal information about executive dysfunctions by eye gaze’s direction, fixations, and brain activity (Scharinger et al., 2015; Bekele et al., 2016). Secondly, several studies showed that data fusion from multiple sensors allowed better analysis and decision since one type’s strengths can compensate for another’s weaknesses (Taher et al., 2015). For example, the eye movements introduce large artifacts to EEG recordings that render data analysis difficult or even impossible (Plöchl et al., 2012). Thus, in standard EEG paradigms, subjects are required to fixate on the screen. A simultaneous recording of eye movements and EEG could allow understanding the artifacts due to the eye movements.

However, to date, very few preliminary studies have integrated both devices to assess executive functioning. For example, Billeci and colleagues used an integrated EEG and eye-tracking approach for the study of responding and initiating joint attention (JA) in children with autism spectrum disorders (Billeci et al., 2017). Specifically, they evaluated the brain function changes and visual pattern simultaneously after 6 months of rehabilitative treatment. Results showed a positive trend of brain activity and connectivity changes after rehabilitative treatment, which were correlated with modifications in gaze measures. Thus, a multimodal approach could characterize JA-related brain circuitries and visual pattern in ASD individuals and monitor longitudinal changes in response to rehabilitative intervention.

### INNOVATIVE 360°-BASED TOOL: EXECUTIVE-FUNCTIONS INNOVATIVE TOOL

**FIGURE 1:**
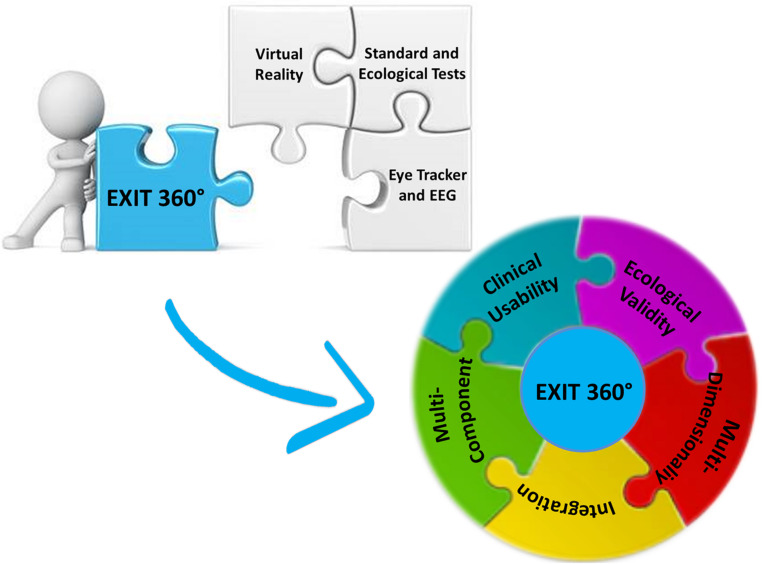
The five pillars behind our innovative 360° assessment tool for EF.

This perspective aims at proposing and discussing the design, creation, and implementation of our 360° Executive-functions Innovative Tool (EXIT 360°), a new 360°-based tool for an innovative assessment of executive dysfunctions (Figure 1). EXIT 360° allows a complete and integrated executive functioning evaluation through an original task for EFs delivered *via* an innovative technological device, a comfortable mobile-powered VR headset combined with ET and EEG sensors. EXIT 360° allows participants to engage in a “game for health,” delivered *via* smartphones, in which they will have to perform several everyday subtasks in 360° daily life environments.

In designing and implementing this evaluation tool, we based on five fundamental concepts for complete and integrated assessment: (1) ecological validity, (2) multicomponent assessment, (3) multidimensionality, (4) integration, and (5) clinical usability (Figure 1).

### EXIT 360°: Ecological Validity

We propose EXIT 360° as an innovative instrument that allows an ecologically valid assessment of executive functioning by implementing everyday scenarios and the chosen subtasks that reproduced daily assignments.

As scenarios, we propose 360° environments that appeared to be a good and engaging setting for the assessment of ecological EFs (Serino et al., 2017; Realdon et al., 2019). The 360° technologies enable participants to be immersed in a real situation from a first-person perspective. Thanks to the mobile-powered VR headset, the subjects can explore the entire space by moving their head, “like in real life” (Serino and Repetto, 2018).

The use of 360° environments allows us to overcome some VR’s limitations. Firstly, the development of 360° environments does not require specialized technological skills or high costs (e.g., expensive software), as they consist of 360° photos or videos that require a standard 360°camera and free online applications (Parsons and Phillips, 2016). Moreover, some studies showed that VR could cause some side effects, such as nausea and dizziness (Parsons, 2015). On the contrary, preliminary studies on PD and MS patients have shown the total absence of adverse events in 360° environments (Serino et al., 2017; Realdon et al., 2019).

**FIGURE 2:**
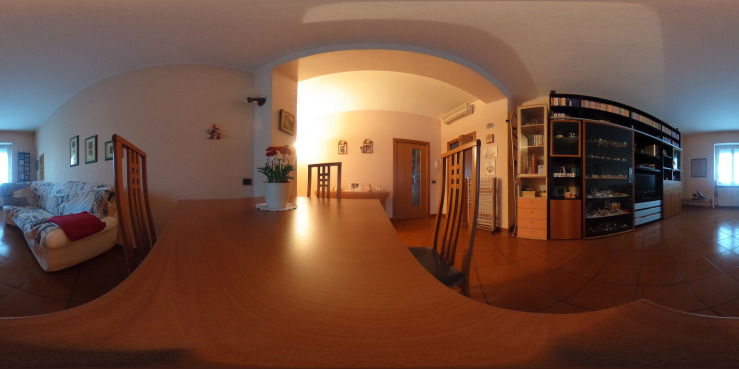
An example of a 360° daily home environment implemented in EXIT 360°.

Unlike previous studies, our environments reproduce everyday domestic setting, such as the kitchen, bedrooms, living room, and landing (Figure 2). Indeed, the VR-based tools for evaluating EFs have always involved several everyday life scenarios such as supermarkets, offices, and schools but fewer domestic environments. In light of the high impact of executive functioning in daily life, we think that an evaluation of the executive impairments in the environment most experienced by the subject could be useful, also with a view to rehabilitation.

In these five environments, the subjects must perform seven subtasks that represent daily life assignments, depending on the environment in which they are immersed. EXIT 360° is profoundly different from other VR-based tools, where participants were immersed in one scenario (e.g., virtual supermarket) and performed only one task (e.g., shopping task). Our tool requires subjects to confront with various everyday situations (e.g., decide what to do if the power goes out in the house).

### EXIT 360°: Multicomponent Assessment

Our innovative EXIT 360° is designed to tap and evaluate different components of executive functioning (multicomponent assessment) simultaneously and quickly.

In EXIT 360°, the participants have the main goal to leave the domestic setting in the shortest possible time. To do this, they must plan a strategy and overcome seven different subtasks of increasing complexity. The idea of implementing these seven steps derives from the desire to create a task that provides a complex evaluation of various components of the EFs. Indeed, each subtask reproduces a common scenario of everyday life that asks subject to perform and successfully pass an assignment that evaluates one or more EFs, according to the instructions and request of each subtask. The subjects will have to respond to subtasks’ requests, choosing between three or more “alternatives” proposed, the one that will allow them to solve the assignment in the best possible way and continue their journey. For example, the first subtask wants to assess the ability of problem-solving and decision-making. The subjects must explore the 360° environment until they see a landing with a closed door that they will have to open to continue the journey. To do this, participants will have to choose among the three alternatives proposed (key, bottle, and screwdriver), the best one to open the door.

The examiner accompanies and guides the participants along the entire path: provides the instructions of the subtasks, collects all verbal answers of the subjects, and manages the transition from one level to another of greater complexity. If the subject chooses a wrong alternative (i.e., does not pass the level), the investigator will have to stop the test immediately.

Overall, EXIT 360° will allow quick, ecologically valid evaluation of possible impairments in seven components of executive functioning: planning, decision-making, problem-solving, attention, visual searching, reasons, and working memory.

Interestingly, EXIT 360° appears as a promising tool usable in evaluating several clinical populations that show various executive dysfunctions. For example, patients with schizophrenia show deficits in planning, mental flexibility, divided attention, and problem-solving. Moreover, an ecologically valid evaluation in contexts similar to everyday scenarios allows overcoming traditional standard assessment limitation. For example, patients with PD showed executive dysfunctions in planning, problem-solving, and flexibility since the early stages of the disease with impairments in everyday life (Maggio et al., 2018). However, some studies showed that traditional standard assessment appeared not sensitive in the early detection of executive deficits (Cipresso et al., 2014). Our tool that will evaluate EFs in contexts similar to everyday life could enable an early identification of these difficulties. This result has a significant impact on PD’s managing since an increasing number of longitudinal studies suggested that early executive dysfunction is predictive of the PD conversion in PD with dementia (Azuma et al., 2003; Janvin et al., 2005). Thus, the early identification of executive deficits could permit identifying patients at risk of developing dementia, providing early neurorehabilitative interventions (Cipresso et al., 2014; Serino et al., 2014).

### EXIT 360°: Multidimensional Assessment

EXIT 360° allows collecting in real time non-verbal information on executive functioning by mobile-powered VR headset in addition to the verbal answer. This innovative technological device integrates the VR headset with eye-tracking cameras and EEG mask with dry electrodes, overcoming the need to invest time and energy into setting up different equipment types.

Specifically, it enables the time-synchronized and automatic acquisition of eye and brain data during an immersive VR experience that involves several executive tasks. These data are transmitted from VR headsets to a desktop application, executable on any computer, *via* USB connection. Thus, researchers can easily check and manage the EEG signals’ quality, the changes in pupil size and position, and the experiments’ progress. All these implicit answers allow us to obtain more information on possible executive dysfunction (Mirsky et al., 2013).

In particular, ET allows detecting any physiological deficits in eye gaze’s direction, saccadic and antisaccadic movements, and fixations by pupil size and position. For example, deficits in antisaccadic activities are recognized as a sensitive marker of frontal lobe impairment (Mirsky et al., 2013). Moreover, the detection of difficulties in eye gaze’s direction and any fixations obtained comparing the points fixed by subjects with those who should have fixed for a correct interpretation of the scenario allows detecting problems in focusing on the most critical components for a correct interpretation of the scene, a specific deficit in patients with executive dysfunction.

Regarding EEG signals, the EEG mask consists of a forehead foam pad with built-in flexible printed circuit board (FPCB) electrodes. The EEG mask involves nine EEG electrodes [AF3, AF4, AF7, AF8, Fp1, Fp2, REF, GND (2)] based on a 10–20 system. Specifically, EEG acquires 6-channel, 24-bit EEG data with sampling rates up to 1 kHz. It allows obtaining data about the electrical activity and any anomalies affecting the prefrontal cortex and related associated cortico-subcortical circuits, responsible for EFs. In line with the literature, possible anomalies in the frontal lobe could explain the executive dysfunctions and any issues in several real-life situations (Baddeley and Wilson, 1988; Burgess et al., 2006).

### EXIT 360°: Integration

The great amount of information received by subjects’ verbal answers and extrapolated from the eye tracker and EEG must be processed and converted into an output.

For the first time, in this tool, we introduce a suitable technique capable of administering a volume of complicated and extensive information: machine learning (ML). Firstly, ML allows constant integration among verbal and non-verbal data. This integration enables our tool to (1) set the device on the individual level (not fixed) and (2) obtain a quicker diagnosis of executive dysfunctions and differentiating in real time the patients according to the clinical group. Two supervised ML algorithms are employed for classification purposes, especially random forest (RF) and support vector machine (SVM). Specifically, RF will allow classifying patients using an ensemble of decision trees, whereas SVM will be used to map inputs to higher-dimensional feature spaces that best separate different classes, minimizing the classification error.

Moreover, it also allows voice recognition of the answers and comparing them with the correct answers to proceed automatically to the test. Finally, the immediate elaboration of data allows not only clinicians to obtain diagnostic information but also patients to gain restitution of what they have done.

### EXIT 360°: Clinical Usability

EXIT 360° is designed to be an innovative 360-based instrument, easily accessible and clinically usable that radically transforms patients’ and the clinicians’ assessment experience. Indeed, EXIT 360° allows in a short time (15/20 min) a complete and integrated evaluation of the EFs with several advantages for clinicians and patients.

Regarding clinicians, EXIT 360° allows them to achieve more information about executive functioning, while participants perform everyday tasks in an environment that reproduce real-life context. In other words, this innovative tool will permit clinicians to directly observe and evaluate the executive impairments that the patients could meet in their own everyday life, overcoming the recall and interviewer biases and neuropsychological tests’ limitations. Moreover, the immediate elaboration of verbal and physiological data allows clinicians to obtain real-time diagnostic information. Additionally, unlike most VR tools, our tool is an easily portable device suitable for clinical settings and for patients with motor difficulties who cannot walk (e.g., initial post-stroke phases). Indeed, compared with other technological devices, such as VIVE, our device is easily transportable allowing clinicians to evaluate patients in their own rooms, overcoming possible difficulty moving. Finally, since our tool does not require special technical skills for use, all healthcare professionals can use it. We think an easy-to-use tool will be used more!

On the other side, Exit 360° allows patients not to undergo a too long and complex evaluation. This tool can be considered a “game for health,” which helps the engagement of patients in an assessment and, at the same time, decreases the anxiety levels typical of neuropsychological evaluation. Moreover, this device allows participants to engage in daily life situation from a first-person perspective while sitting in a safe chair and exploring the entire space just by moving their head. Specifically, patients can proceed with the test only by moving their head, without learning to use technological devices (e.g., joystick) or moving around the environment, with the risk of falling. Finally, ML’s immediate data elaboration allows providing to patients a real-time restitution of what they have done.

## Conclusion

This perspective proposed the design and development of our innovative, interesting, and enjoyable instrument for assessing EFs. The EXIT 360° is a promising 360°-based tool, born to achieve more information about several executive functioning components through an original task for EFs delivered *via* a comfortable mobile-powered VR headset combined with ET and EEG sensors. Specifically, EXIT 360° allows obtaining much clinical information by integrating verbal responses, reaction times, and physiological data of the eye tracker and EEG, also using specific ML’s algorithms. Our tool can be considered an interesting “game for health,” delivered *via* smartphones, in which the participants are engaged in several everyday subtasks in 360° environments that reproduce real-life context.

This innovative 360°-based instrument, easily accessible and clinically usable, will radically transform patients’ and clinicians’ assessment experience. The clinician will get faster, multicomponent, and integrated evaluations. Firstly, EXIT 360° will allow clinicians to collect simultaneously and quickly (15/20 min) data on several components of executive functioning through real-time integration of subjects’ explicit (verbal responses) and implicit (reaction times and physiological data) answers. Moreover, EXIT 360° will allow clinicians to directly collect any executive impairments that could affect the patients’ everyday life and independence since it evaluated them in everyday tasks in environments that reproduce real-life contexts. Our choice is in line with the literature that underlines the importance of evaluating and treating EFs in real-life scenarios due to their key role in everyday life and independent functioning (Chevignard et al., 2000; Fortin et al., 2003; Riva et al., 2020).

On the other side, the patient will be involved in a task that can be experienced as a game, increasing engagement and lowering the level of emotional charge that a neuropsychological evaluation can entail. We think that all participants will evaluate EXIT 360°as usable, engaging, and challenging, and they will show a high sense of presence while immersed in the EXIT 360°. Moreover, this tool will allow participants to receive immediate feedback on their performance due to the immediate data elaboration by ML.

In general, EXIT 360° will allow a quick and ecologically valid evaluation of possible impairments in seven components of executive functioning: planning, decision-making, problem-solving, attention, visual searching, reasons, and working memory. Therefore, EXIT 360° appears as a promising tool in evaluating several clinical populations that show various executive dysfunctions, such as schizophrenia, MS, or PD. For example, a concrete application field of EXIT 360°could be the evaluation of patients with PD where executive dysfunction represents one of the major clinical non-motor symptom in early-stage non-demented PD (Kudlicka et al., 2011; Maggio et al., 2018), particularly disabling due to its high negative impact on daily functioning and quality of life. As said above, traditional standard assessments fail in detecting executive deficits in real-life contexts, especially in the early stage of PD. We suppose that EXIT 360° will be able to reveal different performances in patients with PD compared with HC, in responding correctly to all subtasks, time response, and ability to prone to distractor interference. Moreover, the mobile-powered headset will allow collecting physiological impairments in frontal cortex activation and the antisaccadic eye movements and the number of fixations, typical of PD patients.

Overall, EXIT 360° will allow obtaining simultaneously and in real time more information about executive dysfunction and its impact in real life, allowing clinicians to tailor the rehabilitation to the subject’s needs. Therefore, this tool can be considered a promising ecologically valid instrument for a complete assessment of EFs.

Interestingly, as it was designed, our task could also be used through a streaming platform that would allow the clinicians and patient to carry out remote assessment. This functionality will open up new scenarios for remotely assessing EFs, overcoming the limits of social distancing. Thus, it will allow all patients, included those who cannot reach the clinic, to obtain an evaluation. This is exactly what we want to do!

## Data Availability Statement

The original contributions presented in the study are included in the article/supplementary material, further inquiries can be directed to the corresponding author.

## Author Contributions

FBo and PC developed the new 360° Executive-function Innovative tool. PC, FBa, and GR supervised the sections of Virtual Reality, Eye Tracker, EEG and Machine Learning. EP and FR supervised the section regarding the executive functioning. FBo wrote the manuscript under the final supervision of FBa, EP, FR, GR, and PC. All authors have approved the final version of the manuscript.

## Conflict of Interest

The authors declare that the research was conducted in the absence of any commercial or financial relationships that could be construed as a potential conflict of interest.
